# Impact of leptin deficiency on male tibia and vertebral body 3D bone architecture independent of changes in body weight

**DOI:** 10.14814/phy2.15832

**Published:** 2023-10-03

**Authors:** Alexander Williamson, Alexandre da Silva, Jussara M. do Carmo, Christine L. Le Maitre, John E. Hall, Nicola Aberdein

**Affiliations:** ^1^ Biomolecular Science Research Centre, Department of Bioscience and Chemistry Sheffield Hallam University Sheffield UK; ^2^ Mississippi Center for Obesity Research, Department of Physiology and Biophysics University of Mississippi Medical Center Jackson Mississippi USA

**Keywords:** 3D color coding, density, HFD, micro‐CT, obesity, volume

## Abstract

Leptin an adipokine with potent effects on energy balance and body weight plays an important role in defining bone architecture in growing mammals. However, major changes in body weight can also influence morphology of trabecular and cortical bone. Therefore, we examined the impact of leptin deficiency on tibia and vertebral body 3D bone architecture independent of changes in body weight. Furthermore, advances in computational 3D image analysis suggest that average morphological values may mask regional specific differences in trabecular bone thickness. The study utilized leptin‐deficient Ob/Ob mice (*n* = 8) weight‐paired to C57BL/6 (C57) control mice (*n* = 8) which were split into either lean or obese groups for 24 ± 2 weeks. Whole tibias and L3 vertebrae were fixed before high resolution microcomputed tomography (μCT) scanning was performed. Leptin deficiency independent of body weight reduced tibia cortical bone volume, trabecular bone volume/tissue volume, number, and mineral density. Mean tibia trabecular thickness showed no significant differences between all groups; however, significant changes in trabecular thickness were found when analyzed by region. This study demonstrates that leptin deficiency significantly impacts tibia and vertebral body trabecular and cortical bone 3D architecture independent of changes in body weight.

## 1 INTRODUCTION

Leptin, a potent anorexigenic adipokine secreted by adipocytes, plays an important role in cardiometabolic regulation (Belin de Chantemèle et al., 2009; De Jonghe et al., 2012; do Carmo et al., 2011). Leptin has also been shown to play a role in bone remodeling through the central nervous system (CNS) as well as by its peripheral actions, including direct effects on bone cells (Ducy et al., 2000; Takeda et al., 2002; Turner et al., 2013; Yue et al., 2016). However, it is still controversial whether leptin primarily increases or decreases bone volume and mineral density via peripheral or CNS actions (Ducy et al., 2000; Hamrick et al., 2005; Karsenty & Khosla, 2022; Turner et al., 2013; Yue et al., 2016). Confounding factors that may contribute to this uncertainty include a lack of control for body weight and leptin sensitivity (Bahceci et al., 1999; Bartell et al., 2011; Coleman, 1973; Duan et al., 2007; Grethen et al., 2012). Furthermore, recent advances in 3‐dimensional (3D) microcomputed tomography (μ‐CT) image analysis that may reveal regional differences in bone architecture have not been utilized in previous studies.

Leptin's CNS actions on bone remodeling have been tested using intracerebroventricular (ICV) leptin infusion in leptin‐deficient Ob/Ob mice and have provided contrasting results, with some studies demonstrating a decrease (Ducy et al., 2000) while others show an increase in trabecular bone volume (Bartell et al., 2011; Turner et al., 2013), after leptin infusion. Differences in the dose of leptin administered in these studies may account for some of the variability in bone morphology observed, although each study did observe a reduction in body weight irrespective of leptin dose. Therefore, global leptin deficiency via the ObOb mouse model without the reintroduction of leptin, controlling for body weight, was chosen to reduce these confounding factors in this study.

Iwaniec et al. (2009) evaluated the effect of hypothalamic leptin gene therapy on bone architecture and reported that increased body mass was associated with increased cortical bone volume independent of leptin signaling. However, deletion of leptin receptors on skeletal stem cells increased trabecular bone volume/tissue volume (BV/TV%) suggesting that leptin has non‐CNS actions that reduce bone volume (Yue et al., 2016). In contrast, peripheral leptin delivery in Ob/Ob mice for 2 weeks increased the bone forming surface of cortical endosteum as well as trabecular osteoblast surface and density compared to vehicle treated Ob/Ob mice (Hamrick et al., 2005). Transfer of bone marrow (BM) from leptin‐receptor deficient mice to irradiated wild type (WT) controls also resulted in reduced bone formation rate, compared to WT controls or irradiated WT controls transferred with BM from WT controls (Turner et al., 2013). Many of these apparent inconsistencies may be related to leptin‐mediated changes in body weight which can also influence bone remodeling. Furthermore, most research into the effects of leptin on bone have been completed in murine models. Epidemiological studies on the association of leptin with bone architecture are unclear. The relationship between serum leptin levels and BMD in children aged 11 years old are inconclusive (Kouda et al., 2019). Results often show positive, inverse, or no association between circulating leptin and bone parameters.

Body weight alters bone morphology due to compressive and tensile forces applied to the bone via gravity and skeletal muscle contraction (Greene & Naughton, 2006; Klein‐Nulend et al., 2012). An increase in compression and tensile forces normally leads to increase bone formation, resulting in increased bone volume and/or density (Ducher et al., 2005; Uto et al., 2017; Zhang et al., 2008). However, the impact of obesity and the loading effects of increased body mass index (BMI)/adiposity or eating a high fat diet (HFD) on bone growth and turnover have also proven controversial. Increased BMI has been described as beneficial to bone volume/density and fracture prevention (C De Laet et al., 2005; Dominic et al., 2020; Palermo et al., 2016). Conversely, Zheng et al., (2021) and Driessler and Baldock, (2010) demonstrated that obesity negatively impacted bone, with HFD and central obesity uncoupling bone resorption and formation, leading to increased fracture risk and osteoporosis (Driessler & Baldock, 2010; Zheng et al., 2021). Nevertheless, weight‐pairing in mice has demonstrated that obesity may attenuate the morphological changes in femur and lumbar vertebral bone associated with leptin deficiency (Turner et al., 2014).

The majority of papers reporting on changes in bone architecture over the past decade have conformed to the guidance proposed by Bouxsein et al., in 2010. However, advances in image analysis of μ‐CT data show reporting only mean values may mask underlying regional specific differences in morphology, specifically trabecular thickness between experimental groups. To identify these differences and the effect of leptin deficiency alone on tibia and vertebral bone, this study controlled for changes in body weight by weight‐pairing lean and obese Ob/Ob to lean and obese C57 controls.

## 2 MATERIALS AND METHODS

### 2.1 Mice and treatments

Male C57 (*n* = 8) control mice and male leptin‐deficient Ob/Ob (*n* = 8) mice on the C57BL/6J background were purchased from Jackson Laboratories at 6 ± 2 weeks of age and placed into four groups: lean C57; lean Ob/Ob‐WP; obese C57; obese Ob/Ob‐WP. Mice were placed in individual cages in a 12‐h dark (6 pm to 6 am) and light (6 am to 6 pm) cycle. Mice were given free access to water throughout the study. Leptin‐deficient lean Ob/Ob mice were fed control diet (CD, Harlan Teklad/ENVIGO, CA 8640, energy 3.0 kcal/g, percent kcal from fat (17%), protein (29%), carbohydrate (54%), and food restricted from 6 ± 2 weeks to 24 ± 2 weeks of age to match their weight (weight‐pair WP) with the weight of C57BL/6 (C57) control mice fed CD ad libitum). Leptin‐deficient obese Ob/Ob mice were fed CD, and food intake was controlled from 6 ± 2 weeks to 24 ± 2 weeks of age to match their weight with the weight of C57 control mice HFD ad libitum (HFD, Harlan Teklad/ENVIGO, TD‐0881, energy 4.7 kcal/g, % kcal from fat (44.6%), protein (14.7%), and carbohydrate (40.7%)). Ob/Ob mice body weights were matched to within 5% of C57 lean and obese controls. Food intake and body weight were measured twice a week along with weekly magnetic resonance imaging (4‐in‐1 EchoMRI‐900TM; Echo Medical System) to determine body composition. Mice did not require anesthesia during EchoMRI. No animals, samples, or data were excluded from the reporting.

### 2.2 Food restriction

**FIGURE 1:**
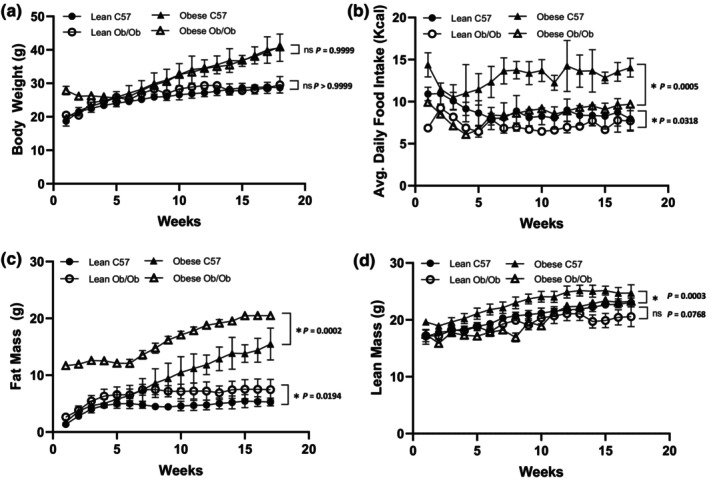
Body composition of male C57 and weight‐paired lean or obese Ob/Ob mice. Lean C57, Lean Ob/Ob, and Obese Ob/Ob were fed a control diet (CD), Obese C57 were fed a high fat diet (HFD). (a). Body weight (g); (b). Average daily food intake in Kcal; (c). Fat Mass (g); (d). Lean mass (g). Mean ± STDev., *n* = 4/group. One‐Way ANOVA or Kruskal–Wallis; *p ≤ 0.05.

Data from literature show Ob/Ob mice eat approximately 6 g chow per day, corresponding to a kcal consumption between 18 and 28 kcal per day depending on constituents of the diet (Skowronski et al., 2017). Consistent weight‐pairing to C57 controls required the Ob/Ob mice to have their daily food intake restricted to approximately 8 kcal per day (Figure 1b). This translated to either 1.7 g/day on the high fat chow (4.7 kcal/g) or 2.6 g/day on the regular chow diet (3 kcal/g). To feed these animals, the high fat chow would constitute a food restriction of over 70% for 24 weeks. We therefore decided on animal welfare grounds that regular chow would be used to avoid undue stress over the extended period of the study. The body composition data show the comparisons in body fat mass and lean mass between the groups.

#### 2.2.1 Tissue harvest

At 24 ± 2 weeks of age, mice were euthanized by excess isoflurane (100%) followed by exsanguination. Both tibiae and L3 lumbar vertebrae were fixed in 4% paraformaldehyde with sodium dihydrogen orthophosphate dehydrate and disodium hydrogen orthophosphate dehydrate for 48 hours before being placed in 70% EtOH until microcomputed tomography (μ‐CT) analysis. For each animal, both tibia were analyzed and the single mean value from both measurements was reported.

#### 2.2.2 Micro‐CT imaging

Samples were removed from 70% EtOH, wrapped loosely in cellophane, mounted into a plastic cylinder, and scanned on a Bruker Skyscan 1272. Scanning parameters include 50 kV X‐ray voltage, 200 μA X‐ray current, 0.5 mm aluminum filter, 4.3–7 μm voxel size with 180° tomographic rotation, and a 0.7° step rotation. Reconstruction was carried out using NRecon software (Bruker Skyscan) and included beam hardening correction of 20% and post‐alignment optimization. Bone mineral density (BMD) was quantified from 2 mm standard calcium hydroxyapatite (CaHA) calibration rods (Bruker Skyscan).

Tibia samples were analyzed in two distinct regions. Firstly, to normalize analysis of cortical bone, measurements were taken exactly 1 mm offset from the bridge break of the growth plate, progressing distally for 1 mm. Secondly, trabecular bone was normalized by measuring 0.2 mm offset from the bridge break of the growth plate and progressing distally for 1 mm. Lumbar vertebrae (L3) sample analysis took place from the first section of distal trabeculae with no primary spongiosa and progressed proximally for 2 mm.

#### 2.2.3 Image post‐processing

Post reconstruction analyses of μCT images were processed in serial slices using CTAn (Bruker Skyscan) software. A normalized region of interest (ROI) was defined by freehand drawing for both cortical and trabecular bone and used during analysis of each tibia and vertebrae throughout the study. Thresholding for cortical BMD was consistent within groups, despeckling white spots was applied to each ROI before 3D analysis was performed.

#### 2.2.4 Quantitative morphometry

Morphometric indexes analyzed in this study were trabecular bone volume fraction (bone volume/ tissue volume BV/TV) %, trabecular thickness (Tb.Th) mm, trabecular number (Tb.N) mm^−1^, trabecular separation (Tb. Sp.) mm, trabecular BMD g/cm^3^, cortical bone volume (BV) mm^3^, cortical bone thickness (mm), and cortical BMD g/cm^3^ as previously described by Bouxsein et al. (2010).

#### 2.2.5 3D color coded bone thickness analysis

Regional trabecular bone thicknesses were analyzed qualitatively using 3D color coding. Trabecular mid‐range thickness values were generated from regions of interest after thresholding and despeckle processing, as outlined above. Contrast stretching in 3D space filtering was applied to the color‐coded datasets, before being modeled in CTvox (Bruker Skyscan). Regional differences in trabecular bone thickness were analyzed qualitatively using a standardized mid‐range transfer function set from minimum 0.00 mm to maximum 0.12 mm. The outer medulla of the trabeculae was partitioned by using the erosion function within CTAn to segregate a 200 μm diameter from the outer most edge of the ROI around the circumference of the ROI. The remaining trabecula inside the original ROI excluding the outer medulla was calculated as the inner core.

#### 2.2.6 3D color coded bone density analysis

Regional specific 3D color‐coded bone density maps were generated in CTVox from original CTAn ROI data sets. Regional differences in cortical bone density were analyzed qualitatively using a standardized mid‐range transfer function and plotted against the limit values of attenuation ranging from minimum 0.0 g.cm^3^ to maximum 2.6 g.cm^3^.

### 2.3 Statistical analysis

Data are presented as mean ± standard deviation or individual data points and mean values. Statistics software GraphPad Prism v8.1.1 was used to test for normality using Shapiro–Wilk normality test. Data were subsequently analyzed using one‐way analysis of variance (ANOVA) with post hoc Tukey test or Kruskal–Wallis with Dunn's Multiple Comparison for three groups or more. Student *t*‐Test and Mann–Whitney for comparison of two groups. A *p* value of ≤0.05 was considered statistically significant. Type II error was calculated using IBM SPSS Statistics v.26, whereby the average observed Power^b^ was calculated as 0.88.

## 3 RESULTS

### 3.1 Leptin deficiency increased fat mass independently of body weight

Food restriction of lean Ob/Ob mice resulted in similar body weights at 24 ± 2 weeks of age compared to lean C57 mice fed CD, 27.55 ± 1.63 g vs 28.58 ± 0.87 g (*p* = 0.3100). Body weight gain was evenly matched throughout the study duration (*p* > 0.9999) (Figure 1a). Similarly, obese Ob/Ob fed CD and obese C57 fed HFD had similar body weights at 24 ± 2 weeks of age, 40.00 ± 0.55 g vs. 42.38 ± 4.08 g (*p* = 0.2927). Body weight gain was evenly matched throughout the study duration (*p* > 0.9999), respectively. The obese C57 and obese Ob/Ob groups had significantly increased body weights at 24 ± 2 weeks of age compared to lean equivalent genotypes, +32.5% and + 31.1%, respectively (*p* = <0.0001), (Figure 1a).

Weight pairing of lean Ob/Ob with lean C57 mice was maintained via food restriction as shown by a significant reduction of 18% in average daily Kcal intake in the Ob/Ob group (*p* = 0.0318), (Figure 1b). Obese Ob/Ob consumed on average 34% less Kcal per day to maintain body weight of weight matched obese C57 controls (*p* = 0.0005) (Figure 1b). Lean Ob/Ob had significantly higher fat mass (*p* = 0.0194) but similar lean mass (*p* = 0.0768) compared to lean C57 mice over the duration of the study (Figure 1c,d). Obese weight‐paired Ob/Ob mice had significantly increased fat mass (*p* = 0.0002) and significantly less lean mass (*p* = 0.0003) compared to obese C57 controls consuming a HFD (Figures 1c,d).

### 3.2 Leptin deficiency reduced tibia cortical bone volume independently of body weight

**FIGURE 2:**
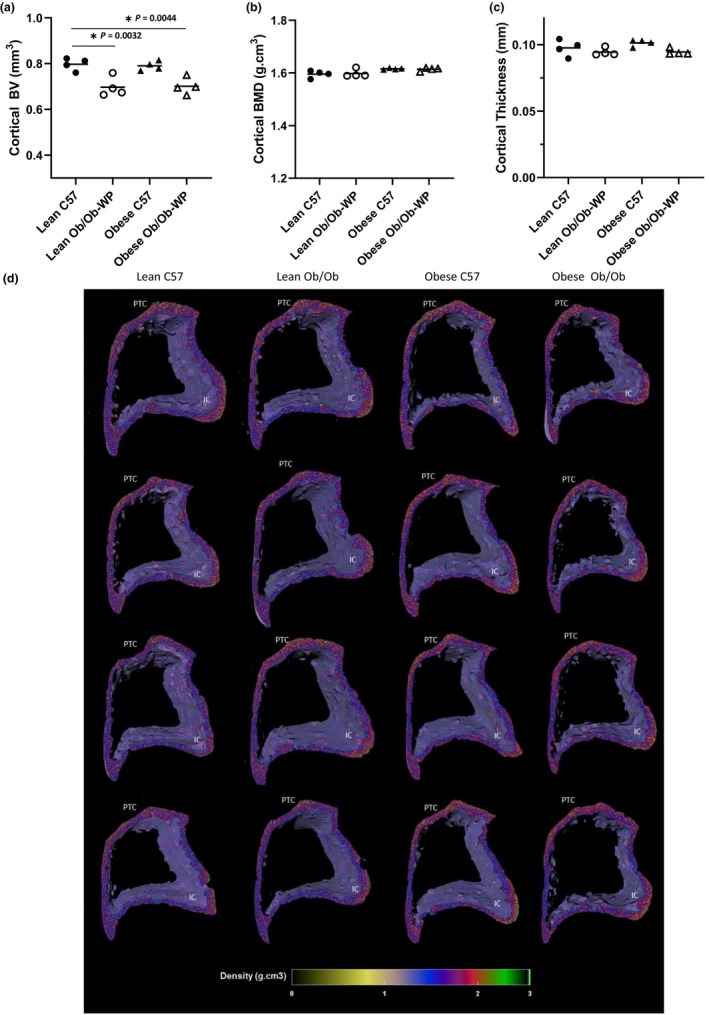
Proximal tibia cortical bone morphology at 24 ± 2 weeks of age of male C57 and weight‐paired lean or obese Ob/Ob mice. (a). Cortical bone volume (BV) (mm^3^); (b). Cortical bone mineral density (BMD) (g/cm^3^); (c). Cortical Thickness (mm); (d). 3D color‐coded density map (g.cm^3^) showing increased density at interosseous crest (IC) and proximal tibial crest (PTC). *n* = 4 Mean and individual data points. One‐way ANOVA or Kruskal–Wallis with post hoc multiple comparisons, *p ≤ 0.05 vs. Lean C57.

Tibia cortical BV was significantly reduced in lean Ob/Ob (*p* = 0.003) and obese Ob/Ob (*p* = 0.004) mice when compared to lean C57 controls, whereas obese C57 mice exhibited similar (*p* = 0.989) tibia cortical BV compared to lean C57 controls (Figure 2a). Tibia cortical BMD was unaffected by leptin deficiency or increased body weight compared to lean C57 controls (Figure 2b). Overall tibia cortical thickness was also unaffected by leptin deficiency or increased body weight compared to controls (Figure 2c). Qualitative regional increases in tibia cortical BMD (g.cm^3^) were mapped to areas associated with compression loading including the interosseous crest (IC) and the proximal tibia crest (PTC) (Figure 2d).

### 3.3 Leptin deficiency and HFD reduced tibia trabecular bone number, bone mineral density, and increased trabecular separation

**FIGURE 3:**
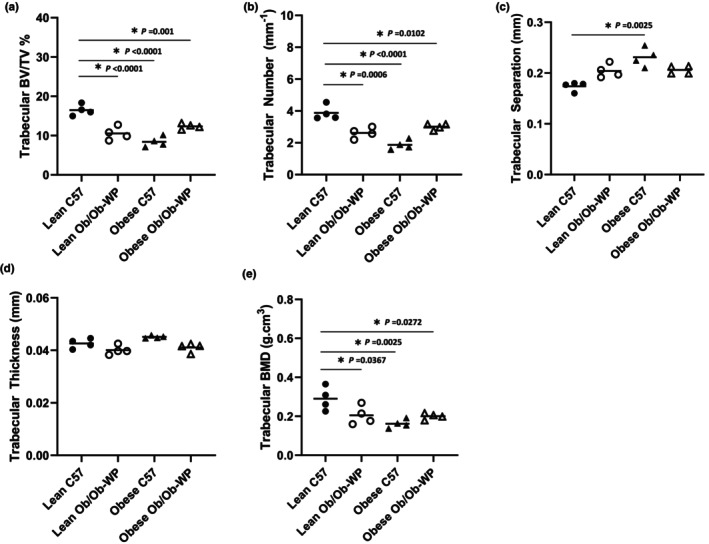
Proximal tibia trabecular bone morphology at 24 ± 2 weeks of age of male C57 and weight‐paired lean or obese Ob/Ob mice. (a). Trabecular bone volume/tissue volume (BV/TV) (%); (b). Trabecular number (mm^−1^); (c). Trabecular separation (mm); (d). Trabecular thickness (mm); (e). Trabecular bone mineral density (BMD) (g/cm^3^). *n* = 4 Mean and individual data points. One‐way ANOVA or Kruskal–Wallis with post hoc multiple comparisons, *p ≤ 0.05 vs. Lean C57.

HFD‐fed obese C57 mice exhibited the greatest reduction in tibia trabecular BV/TV% (*p* < 0.0001) and BMD (g.cm^3^) (*p* = 0.0025) compared to lean C57 controls (Figure 3a,e). Lean and obese Ob/Ob mice also exhibited 35% and 29% reductions in trabecular BV/TV%, respectively, compared to lean C57 control mice (*p* < 0.0001 and *p* = 0.001, respectively) (Figure 3a). Leptin deficiency also significantly reduced BMD compared to lean C57 controls (Lean Ob/Ob vs. Lean C57 *p* = 0.0367; obese Ob/Ob vs. Lean C57 *p* = 0.0272) (Figure 3e). The changes in bone volume appear to be due to a significant reduction in trabecular number (Lean Ob/Ob vs. Lean C57 *p* = 0.0006; obese C57 vs. Lean C57 *p* < 0.0001; obese Ob/Ob vs. Lean C57 *p* = 0.0102) (Figure 3b) and proportionate increase in trabecular separation (Lean Ob/Ob vs. Lean C57 *p* = 0.2629; obese C57 vs. Lean C57 *p* < 0.0025; obese Ob/Ob vs. Lean C57 *p* = 0.1128) (Figure 3c). Leptin‐deficient or HFD‐fed C57 mice did not significantly reduce mean trabecular thickness compared to lean C57 controls (Lean Ob/Ob vs. Lean C57 *p* = 0.1167; obese C57 vs. Lean C57 *p* < 0.1167; obese Ob/Ob vs. Lean C57 *p* = 0.1868) (Figure 3d).

### 3.4 Regional differences in tibia trabecular thickness distribution are masked by overall thickness mean

**FIGURE 4:**
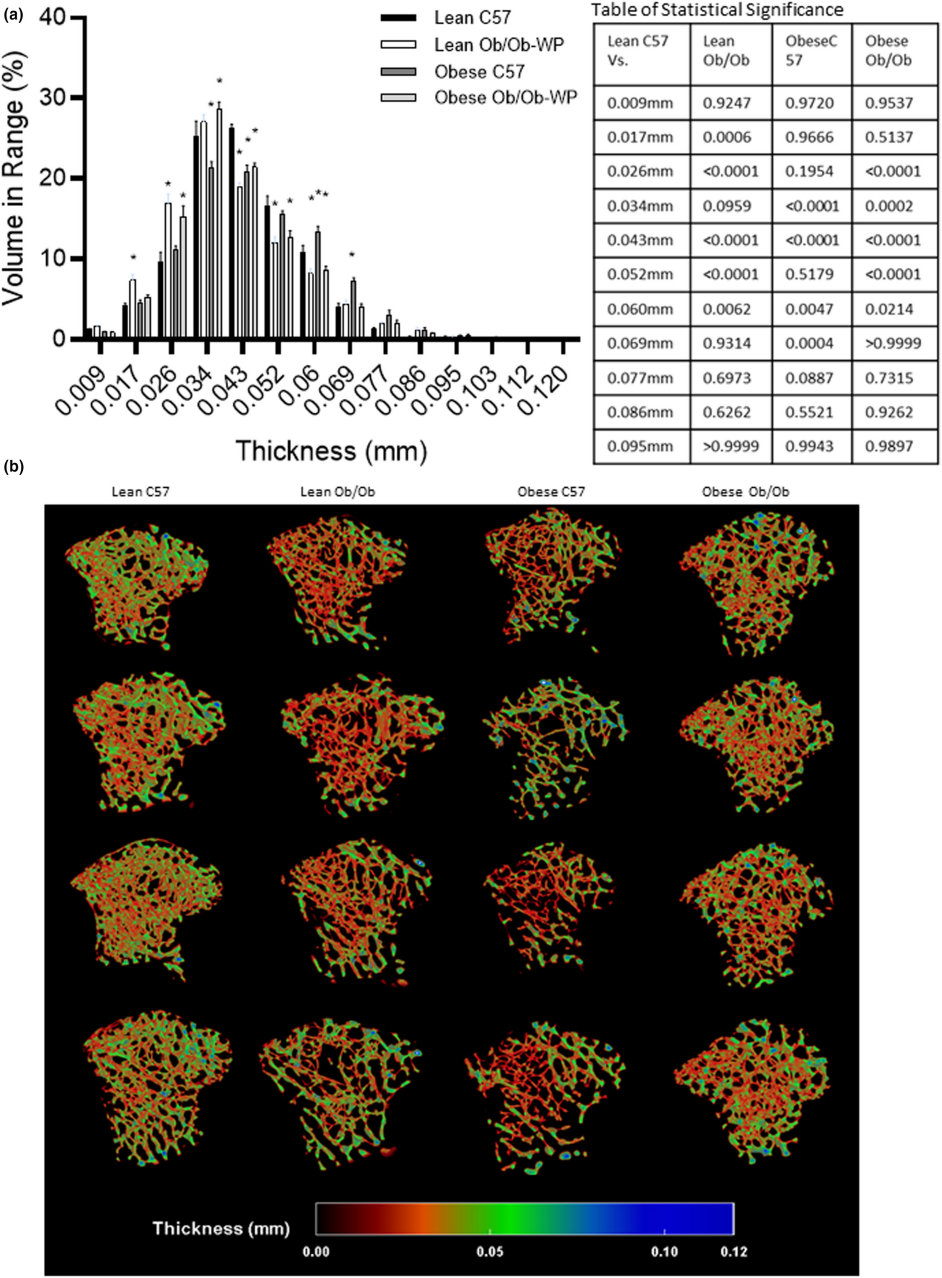
Proximal tibia trabecular bone morphology at 24 ± 2 weeks of age of male C57 and weight‐paired lean or obese Ob/Ob mice. (a). Thickness distribution (mm) plus table of statistical significance; (b). 3D color‐coded thickness map (mm). *n* = 4 Mean and individual data points. One‐way ANOVA or Kruskal–Wallis with post hoc multiple comparisons, *p ≤ 0.05 vs. Lean C57.

**FIGURE 5:**
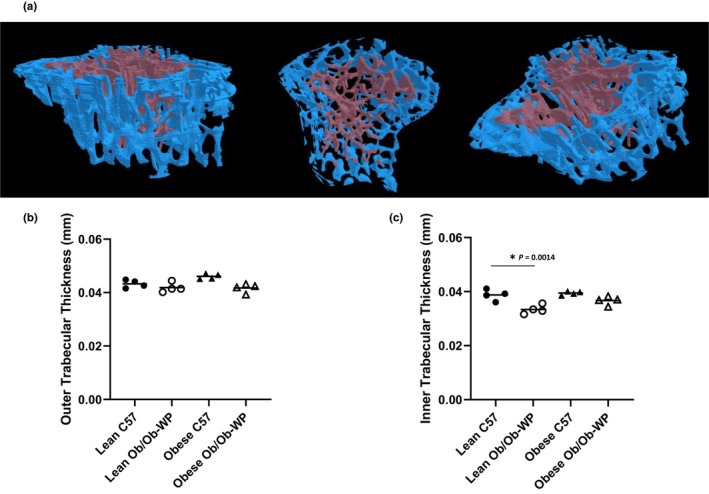
Inner vs. Outer proximal tibia trabecular bone morphology at 24 ± 2 weeks of age of male C57 and weight‐paired lean or obese Ob/Ob mice. (a). Representative model of inner and outer medullar trabecular bone region of interest; (b). Outer medullar trabecular thickness (mm); (c). Inner trabecular thickness (mm). *n* = 4 Mean and individual data points. One‐way ANOVA with post hoc multiple comparisons, *p ≤ 0.05 vs. Lean C57.

A qualitative 3D color‐coded thickness transfer function from 0.009 to 0.112 mm was developed and applied to demonstrate the distribution of tibia trabecular thickness across the entire 3D ROI. Although leptin deficiency and HFD‐induced increases in body weight were not associated with any overall difference in mean trabecular thickness compared to lean C57 control mice (Figure 3d), the qualitative 3D color‐coded thickness maps revealed regional specific changes in thickness distribution and maximum thickness values across all groups (Figure 4b). Lean Ob/Ob mice exhibited a significant increase in percentage of thinner struts (0.017–0.026 mm, *p* < 0.0006) and a significant reduction in the percentage of mid‐range to thicker struts (0.043–0.060 mm, *p* < 0.0062) compared to lean C57 controls. Obese C57 mice exhibited a significant reduction in percentage of mid‐range struts (0.034–0.043 mm, *p* < 0.0001) and a significant increase in the percentage of thicker struts (0.060–0.069 mm, *p* < 0.0047) compared to lean C57 controls. Obese Ob/Ob mice closely matched the distribution pattern of lean Ob/Ob mice whereby obese Ob/Ob exhibited a significant increase in thinner struts (0.026–0.034 mm, *p* < 0.0002) and a significant reduction in mid‐range to thicker struts (0.043–0.060 mm, *p* < 0.0214) compared to lean C57 controls (Figure 4a). Furthermore, trabeculae were segmented in to two section, an inner core and outer medulla for region specific analysis (Figure 5a). Trabecular thickness of the inner core region was significantly reduced in the Lean ObOb compared to the lean C57 control mice, Figure 5c (*p* = 0.0014); however, the outer regions across all groups were unchanged (Figure 5b).

### 3.5 Leptin deficiency reduced vertebral cortical bone thickness

**FIGURE 6:**
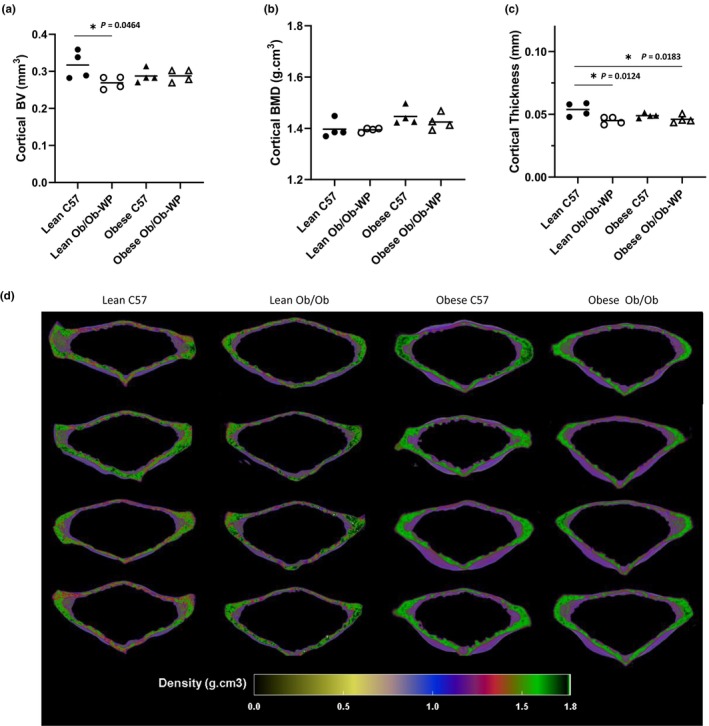
Vertebral cortical bone morphology at 24 **±** 2 weeks of age of male C57 and weight‐paired lean or obese Ob/Ob mice. (a). Cortical bone volume (BV) (mm^3^); (b). Cortical bone mineral density (BMD) (g/cm^3^); (c). Cortical Thickness (mm); (d). 3D color‐coded density map (g.cm^3^) of vertebral body cortical bone. *n* = 4 Mean and individual data points. One‐way ANOVA or Kruskal–Wallis with post hoc comparisons, *p ≤ 0.05 vs. Lean C57.

Leptin deficiency in lean mice significantly reduced vertebral cortical bone volume compared to lean C57 controls (*p* = 0.0464) (Figure 6a). However, body weight did not significantly alter overall vertebral cortical bone volume. Similarly, vertebral cortical BMD was unchanged in leptin‐deficient Ob/Ob mice or HFD‐fed C57 mice compared to lean controls (Figure 6b). Cortical thickness, however, was reduced by leptin deficiency independent of increased body weight as both lean Ob/Ob (*p* = 0.0124) and obese Ob/Ob (*p* = 0.0183) mice had significantly reduced mean vertebral cortical thickness compared to lean C57 controls (Figure 6c). Qualitative regional 3D color maps of BMD demonstrated a similar distribution pattern across all four groups (Figure 6d).

### 3.6 High fat diet reduced vertebral trabecular bone volume, number, and mineral density

**FIGURE 7:**
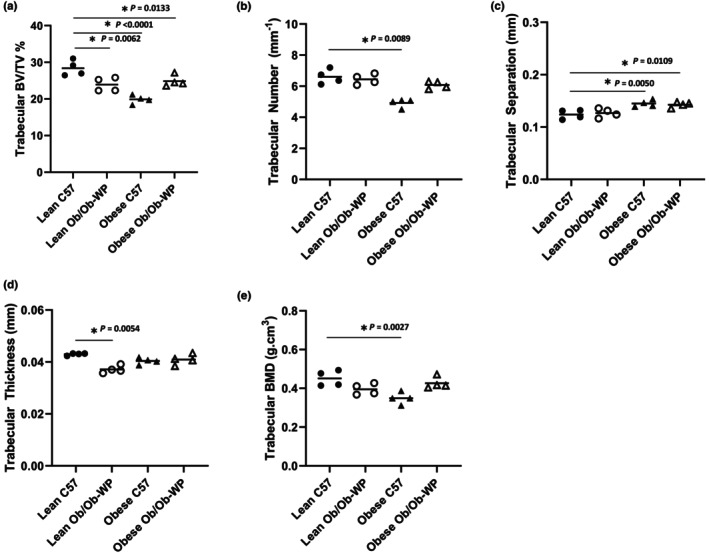
Vertebral trabecular bone morphology at 24 **±** 2 weeks of age of male C57 and weight‐paired lean or obese Ob/Ob mice. (a). Trabecular bone volume/tissue volume (BV/TV) (%); (b). Trabecular number (mm^−1^); (c). Trabecular separation (mm); (d). Trabecular thickness (mm); (e). Trabecular bone mineral density (BMD) (g/cm^3^). *n* = 4 Mean and individual data points. One‐way ANOVA or Kruskal–Wallis with post hoc comparisons, *p ≤ 0.05 vs. Lean C57.

Leptin deficiency and HFD significantly reduced vertebral trabecular BV/TV% (Figure 7a). However, only obese C57 mice fed a HFD had significant reductions in both trabecular number (Figure 7b) (*p* = 0.0089), mineral density (Figure 7e) (*p* = 0.0027), and a complementary increase in trabecular separation (Figure 7c) (*p* = 0.0050). Reductions in lean Ob/Ob vertebral bone volume were associated with reductions in trabecular thickness (Figure 7d) rather than number (Figure 7b). Reductions in vertebral bone volume of obese Ob/Ob were associated with small but insignificant reductions in both vertebral trabecular number and thickness, which resulted in a significant increase in vertebral trabecular separation (Figure 7c).

### 3.7 Regional differences in vertebral trabecular thickness distribution are masked by overall thickness mean

**FIGURE 8:**
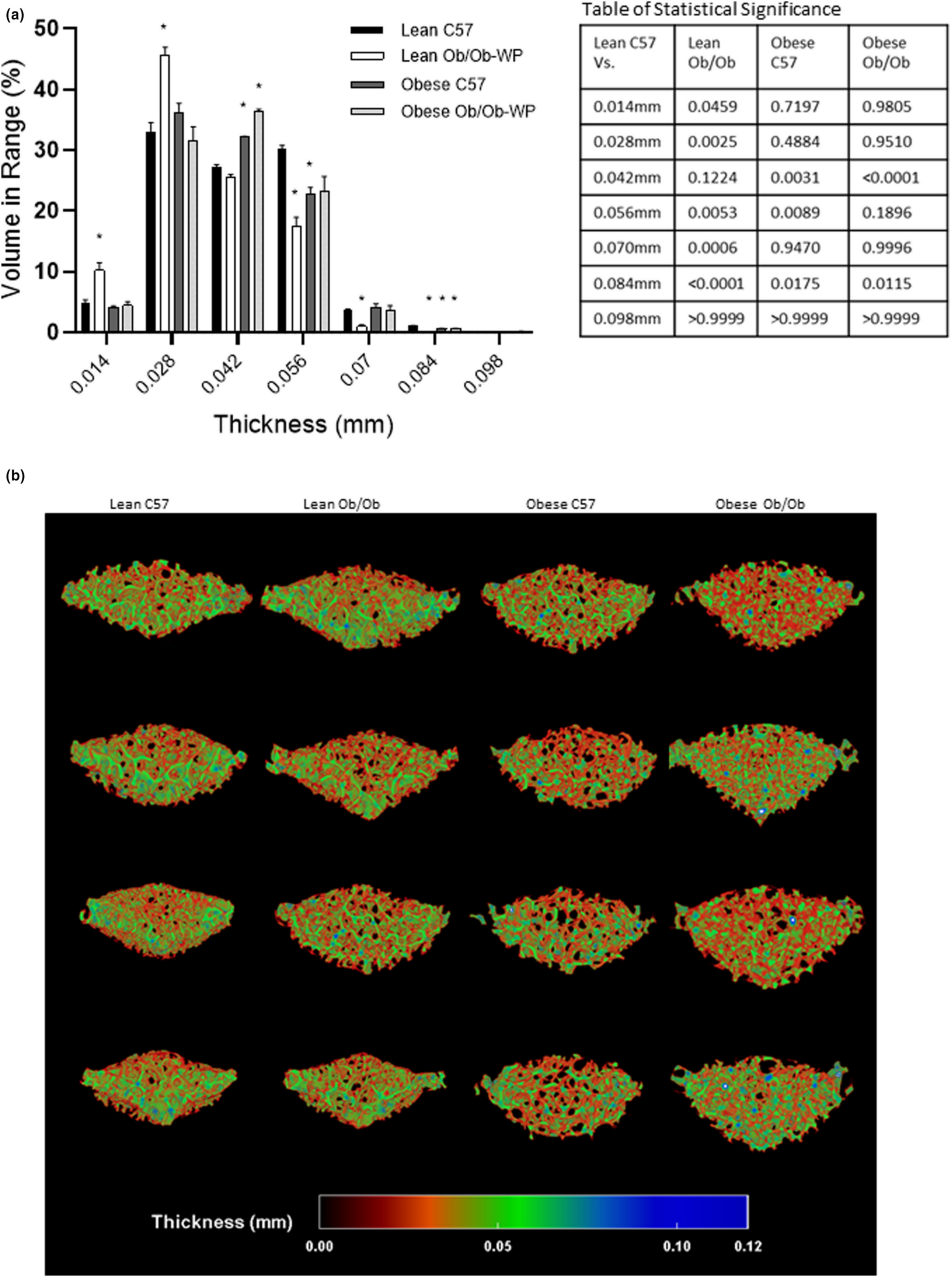
Vertebral trabecular bone morphology at 24 ± 2 weeks of age of male C57 and weight‐paired lean or obese Ob/Ob mice. (a). Thickness distribution (mm) plus table of statistical significance; (b). 3D color‐coded thickness map (mm). *n* = 4 Mean and individual data points. One‐way ANOVA or Kruskal–Wallis with post hoc multiple comparisons, *p ≤ 0.05 vs. Lean C57.

A qualitative 3D color‐coded thickness transfer function from 0.009 to 0.103 mm was developed and applied to examine the distribution of vertebral trabecular thickness across the entire 3D ROI. Although lean Ob/Ob mice were the only group to show a significant reduction in trabecular thickness compared to lean C57 control mice (Figure 7d), the 3D color maps (Figure 8b) revealed changes in thickness distribution and maximum thickness values across all groups. Lean Ob/Ob mice exhibited a significant increase in percentage of thinner struts (0.014–0.028 mm, *p* < 0.0459) and a significant reduction in the percentage of thicker struts (0.056–0.070 mm, *p* < 0.0053) compared to lean C57 controls. Both obese C57 and obese Ob/Ob mice closely matched the percent distribution of thin and thick trabecular compared to lean C57 mice. However, both obese groups exhibited a significant increase in the percentage of mid‐range struts at 0.042 mm (Obese C57 *p* = 0.0031; obese Ob/Ob *p* < 0.0001) compared to lean C57 controls (Figure 8a).

## 4 DISCUSSION

### 4.1 Effects of leptin deficiency on cortical and trabecular bone morphology

Previous studies have provided contradictory results on the role for leptin on bone morphology in vivo. However, few studies have accounted for the changes in body weight associated with the anorexigenic effects of leptin. We conducted a long‐term in vivo investigation using a mouse model of leptin deficiency (Ob/Ob) and weight‐paired them to lean and obese C57 controls, to better understand the role of leptin on bone morphology independent of changes in body weight.

The current study demonstrates that leptin deficiency, independent of changes in body weight, reduced tibia cortical bone volume, trabecular BV/TV%, number, and mineral density. We also found that assessment of average changes in trabecular thickness masked significant differences in the region and size distribution of trabeculae within the tibia.

An extensive review by Reid et al. (2018) detailed the effects of leptin and leptin deficiency in Ob/Ob mice on the skeleton including tibia and vertebrae. However, most data included were published before 2013, and in most instances, the impact of differences in body weight due to leptin deficiency or leptin administration was not determined.

Previous studies have shown that reductions in overall tibia and vertebral body bone volume were closely linked to increased bone marrow adipose tissue (BMAT) (Costa et al., 2019; Suresh et al., 2020). BMAT has been suggested to play a role in regulating the trabeculae of long bones of the femur and tibia with numerous studies suggesting important interactions between red marrow adipocytes and bone cell differentiation. As both osteoblasts and red marrow adipocytes differentiate from the same pluripotent mesenchymal stem cell (MSC) lineage, there is evidence to suggest a competitive relationship (Hamrick et al., 2005; Yue et al., 2016). Leptin has been shown to affect bone turnover by acting directly on bone cells, producing an anabolic effect on osteoblasts, while inhibiting osteoclastogenesis (Reid et al., 2018). Treatment of primary osteoblasts with leptin has also been shown to cause dose‐dependent increases in in vitro mineralization and alkaline phosphatase (ALP) activity (Reseland et al., 2001). Similarly, early evidence suggested that leptin signaling enhanced the differentiation of marrow stromal cells into osteoblast lineage, while suppressing adipogenic differentiation (Thomas et al., 1999). In recent years, conflicting evidence has suggested that leptin signaling promotes adipogenesis while suppressing osteogenesis in mesenchymal stromal cells and that a high fat diet promotes adipogenesis through LepR signaling in bone marrow stromal cells (Yue et al., 2016). Results from the current study are consistent with the hypothesis that leptin deficiency may reduce osteoblast differentiation, promoting adipogenesis in the red marrow; however, histological analysis would be needed to assess this potential mechanism.

### 4.2 Effects of high fat diet on cortical and trabecular bone morphology

In the vertebral body, reductions in BMD were only significant in the HFD‐induced obese C57 mice compared to lean C57 controls. This finding is consistent with those of Patsch et al. (2011) who demonstrated reductions in vertebral trabecular BMD in response to both short‐ and long‐term HFD‐induced obesity. These results are despite differences in sample collection including analysis of long vs. short bones, 2D histological vs. 3D μCT analysis and the level of resolution of μCT imaging 10.5 μm vs. 7 μm resolution. Cao et al. (2009) demonstrated a significant reduction in BV/TV% in C57 mice fed a HFD compared to controls, similar to results of the current study. However, Cao et al. (2009) did not determine the independent contribution of body weight or leptin. Furthermore, obese C57 mice exhibited significant reductions in trabecular BV/TV% compared to lean C57 controls. This result supports the findings of Montalvany‐Antonucci et al. (2018) who demonstrated that a HFD decreased femur trabecular BV/TV% by approximately 50% compared to controls.

Leptin's role in controlling bone morphology in HFD‐induced obese subjects is further complicated by the possibility that increased adiposity may lead to leptin resistance in the CNS and in peripheral tissues. Leptin resistance may occur in obese individuals with high circulating leptin levels, and potential mechanisms involved have been covered in detail previously (Sáinz et al., 2015). Some studies suggest that leptin resistance in obesity may be selective with an attenuation of many of the metabolic responses to leptin but preserved effects to activate the sympathetic nervous system and to raise blood pressure (do Carmo et al., 2013; Hall et al., 2019). Whether obese C57 male mice in the current study were affected by leptin resistance, perhaps explaining some of the similarities with obese Ob/Ob mice bone morphometry, requires further investigation. Furthermore, whether the effects could be mediated via direct (MSCs) or indirect (CNS) mechanisms is unclear and beyond the scope of this investigation.

### 4.3 Effects of load on cortical and trabecular bone morphology

Lean mass and fat mass are believed to independently contribute to bone remodeling and have positive correlations with increased compressive force of the knee joint in obese individuals (Messier et al., 2014). In humans, an increase in lean mass is associated with a greater increase in whole body BMD (mg.cm^2^) (Zhu et al., 2015), whereas low muscle mass positively correlate with low BMD (Hamrick et al., 2004). These data suggest that lean mass is a better predictor of BMD than fat mass. However, the current study demonstrated that obese groups with 30% higher overall body mass and a significant increase in % fat and % lean mass had a significant reduction in tibia trabecular BMD and no change in cortical BMD compared to lean C57 controls. A study of 224 women also recently demonstrated that percentage fat mass is negatively associated with whole body BMD (Engberg et al., 2020). Furthermore, prepubertal obese children have a significant decrease in whole body BMD and bone mineral content (BMC) when compared to lean controls after normalizing for lean mass (Rocher et al., 2008). Studies of independent cohorts of adolescent children also show that adiposity is inversely correlated with BMD in specific bone regions (Hong et al., 2010; Pollock et al., 2007). These data suggest that fat mass may be inversely correlated with trabecular BMD but not cortical in the tibia of mice.

Tibia and vertebral body cortical and trabecular bone volume was consistently reduced in the obese groups compared to control. This observation is consistent with findings of other groups who demonstrated that bone volume is not positively correlated with increases in load. Cao et al. (2009) demonstrated that increased body weight due to HFD did not significantly increase tibia cortical bone area (mm^2^) in C57 mice compared to control mice fed a CD. Furthermore, Lorincz et al. (2010) when adjusting for body mass demonstrated cortical BV (mm^2^/g) was actually reduced in high fat, high sucrose fed C57 mice with increased body weight. Turner et al. (2013) demonstrated, via histomorphometry, that trabecular femur epiphysis BV (%) was similar in Ob/Ob and wild type controls despite a significant difference in body weight. This finding agrees with the current study which demonstrates that increased overall body weight due to leptin deficiency or HFD did not increase cortical bone volume (mm^3^) in either C57 or Ob/Ob mice, and in fact, a significant decrease in BV/TV% was observed in tibia and vertebral body trabeculae. Although Cao et al. analyzed a smaller trabecular bone ROI and at a different offset from the bridge break, they identified trabecular BV/TV% and number were decreased in mice fed a HFD, and trabecular separation was increased. Iwaniec et al. (2009) found that increased body mass due to HFD, as opposed to leptin gene therapy, was responsible for an increase in mid‐shaft femur cortical BV, and thickness. These findings suggest that leptin deficiency independent of body weight partially account for the adverse effects of HFD on bone morphometry, and that increased body weight may ameliorate some of the negative effects of leptin deficiency alone.

### 4.4 Trabecular thickness and clinical implications

Consistent with the results of Cao et al. (2009), the current study demonstrated that average trabecular thickness remained unchanged in response to leptin deficiency independent of load or diet. However, visual inspection of the 3D data highlighted spatial differences in the thickness of core trabeculae compared to trabecular medulla. Technological advancement in μCT imaging/analysis in the last 10 years, including the 3D spatial reconstruction of cortical and trabecular thickness and density, has allowed for the visualization of regional specific changes in morphology. The current study has been able to categorize the trabeculae in 3D in a way that has previously not, to our knowledge, been described in this model. Kerckhofs et al. (2016) demonstrated a similar system of 3D color mapping of tibia trabecular thickness in a HFD model of type 2 diabetes mellitus and found an overall increase in trabecular thickness and no change in BV/TV%, or separation in their model.

Knowledge of the regional and spatial changes associated with trabeculae in particular is important as links between fracture risk and trabecular thinning have been well established in humans (McCloskey et al., 2016; Osterhoff et al., 2016; Tamura et al., 2004). For example, vertebral trabecular bone score (TBS) in both men and women was a significant independent predictor of fracture (C. De Laet et al., 2005). Furthermore, Fields and Keaveny (2012) summarized that trabecular architecture, most notably a reduction in vertical struts, increased vertebral fragility. They also concluded that increasing knowledge of regional specific changes in trabeculae is important to improve fracture risk predictions in a clinical setting. Femoral neck fracture risk may also be reduced if interventions focus on increasing proximal femur trabecular bone architecture (Thomas et al., 2009). Obesity and percentage fat accumulation have been linked with all incidence fracture risk in older adults more so than BMI (Gandham et al., 2020). However a recent meta‐analysis of fracture risk in obese adults described the need for site specific fracture studies, specifically differences in bone microarchitecture in models with and without obesity (Turcotte et al., 2021).

### 4.5 Confounding factors and limitations of the study

It should be noted that the obese Ob/Ob mice were fed a CD instead of the HFD‐fed to the obese C57 controls in the interest of animal welfare and this could be considered a limitation of the study. However, to avoid this potential confounding factor, obese C57 bones were not statistically compared to obese ObOb bones, only to lean C57 controls. Nevertheless, the obese Ob/Ob mice ate a different profile of dietary fats and carbohydrates compared to the obese C57 group.

HFD, defined as a diet from which >30% of energy intake is accounted for by lipid consumption, has been reported to exert an effect upon bone, promoting bone loss and osteoporosis (Qiao et al., 2021). HFD‐fed mice are often used to model obesity‐induced bone loss (Silva et al., 2019). However, the independent effects of HFD consumption on bone remodeling are difficult to separate from the concomitant effects of HFD‐induced increases in mechanical load from body weight, serum leptin, leptin resistance, and body fat percentage (Mendoza‐Herrera et al., 2021). The possibility that the obese C57 mice fed the HFD may become leptin resistant was considered due to the similar response in measured parameters to the obese Ob/Ob mice. It is well established that adipose tissue depots are positively correlated with circulating plasma leptin levels (Lönnqvist et al., 1997) and both obese C57 and obese Ob/Ob exhibited significant increases in overall body fat mass, compared to control mice.

In addition, HFD promotes adipogenic differentiation of MSCs and yellow bone marrow formation, in turn suppressing osteoblast differentiation (Qiao et al., 2021). Co‐culture of preosteoclasts with adipocytes has been shown to increase the presence of TRAP‐positive multinucleated osteoclasts in vitro (Montalvany‐Antonucci et al., 2018). HFD‐induced links to oxidative stress (OS) may also play a role. In brief, the exact relationship between OS and HFD consumption on bone remodeling remains poorly defined. However, it is known that HFD contributes to oxidative stress via production of reactive oxygen species (ROS) (Tan & Norhaizan, 2019) and ROS have been shown to activate osteoclast differentiation while inducing osteocyte apoptosis and inhibiting osteoblast activity, facilitating bone resorption and suppressing bone formation (Domazetovic et al., 2017). Similarly, HFDs induce metabolic inflammation in many organs, increasing levels of endotoxins, hormones (growth hormone//insulin‐like growth factor‐1 and elevated parathyroid hormone) circulating free fatty acids and inflammatory mediators (Duan et al., 2018). There are clearly many links between HFD feeding and bone turnover that are beyond the scope of this study but have been largely controlled for by maintaining total body weight and comparing body composition in the form of fat and lean mass.

In conclusion, leptin deficiency independent of changes in body weight had a significant effect on both tibia and vertebral cortical and trabecular bone *in vivo*. The overall morphological changes were similar in size and effect to that of HFD‐induced obesity. However, technological advances in image analysis of μCT data have allowed for previously hidden changes in regional and spatial trabecular thickness to be quantified using 3D spatial segmentation. Further analysis using this segmentation approach may allow clinicians to better understand overall fracture risk and more accurately help identify preventative measures in at risk groups.

## AUTHOR CONTRIBUTIONS

This work was carried out in the Aberdein laboratory within the Biomolecular Science Research Centre, Department of Bioscience and Chemistry, Sheffield Hallam University, UK and the Hall laboratory, Mississippi Center for Obesity Research, Department of Physiology and Biophysics, University of Mississippi Medical Center, MS. The authors listed in parenthesis contributed to the following aspects of the study: Conception or design of the work. (Nicola Aberdein, Alexander Williamson, Christine L Le Maitre, John E Hall). Acquisition, analysis, or interpretation of data for the work. (Nicola Aberdein, Alexander Williamson, Christine L Le Maitre, John E Hall, Alexandre da Silva, Jussara M do Carmo). Drafting of the work or revising it critically for important intellectual content. (Nicola Aberdein, Alexander Williamson, Christine L Le Maitre, John E Hall, Alexandre da Silva, Jussara M do Carmo). Approved the final version of the manuscript. (Nicola Aberdein, Alexander Williamson, Christine L Le Maitre, John E Hall, Alexandre da Silva, Jussara M do Carmo). Agree to be accountable for all aspects of the work in ensuring that questions related to the accuracy or integrity of any part of the work are appropriately investigated and resolved. (Nicola Aberdein, Alexander Williamson, Christine L Le Maitre, John E Hall, Alexandre da Silva, Jussara M do Carmo). All persons designated as authors qualify for authorship, and all those who qualify for authorship are listed. (Nicola Aberdein, Alexander Williamson, Christine L Le Maitre, John E Hall, Alexandre da Silva, Jussara M do Carmo).

## CONFLICT OF INTEREST STATEMENT

The authors do not have any conflict of interest.

## ETHICS STATEMENT

This study was approved by the Institutional Animal Care and Use Committee (IACUC) of the University of Mississippi Medical Center, Jackson, MS (Approval Number 1154C/1154D). All animal experiments followed the Guide for the Care and Use of Laboratory Animals (2011 Eighth Edition, National Research Council) for the welfare of the laboratory animals. All necessary procedures were implemented to minimize the pain and suffering of animals. This included the avoidance of unnecessarily harsh food restriction regimes using high fat diet, instead feeding obese Ob/Ob mice a standard chow diet. No animals, samples, or data were excluded from the reporting.

## Data Availability

The datasets used and/or analyzed during the present study are stored in the Sheffield Hallam SHURA data repository and can be made available by contacting the corresponding author upon reasonable request.
